# Emerging roles for the nucleolus in development and stem cells

**DOI:** 10.1242/dev.204696

**Published:** 2025-05-14

**Authors:** Bryony J. Leeke, Imke Staffhorst, Michelle Percharde

**Affiliations:** ^1^ MRC Laboratory of Medical Sciences, London W12 0HS, UK; ^2^ Institute of Clinical Sciences, Imperial College London, London W12 0HS, UK

**Keywords:** Nucleoli, Development, Heterochromatin, ESCs, NADs, Nuclear architecture

## Abstract

The nucleolus is a membrane-less subnuclear compartment known for its role in ribosome biogenesis. However, emerging evidence suggests that nucleolar function extends beyond ribosome production and is particularly important during mammalian development. Nucleoli are dynamically reprogrammed post-fertilisation: totipotent early mouse embryos display non-canonical, immature nucleolar precursor bodies, and their remodelling to mature nucleoli is essential for the totipotency-to-pluripotency transition. Mounting evidence also links nucleolar disruption to various pathologies, including embryonic lethality in mouse mutants for nucleolar factors, human developmental disorders and observations of nucleolar changes in disease states. As well as its role in ribogenesis, new findings point to the nucleolus as an essential regulator of genome organisation and heterochromatin formation. This Review summarises the varied roles of nucleoli in development, primarily in mammals, highlighting the importance of nucleolar chromatin for genome regulation, and introduces new techniques for exploring nucleolar function.

## Teaser

**Summary:** This Review explores roles of the nucleolus in mammalian development, with a particular focus on genome organisation and chromatin regulation.

## Introduction

The nucleolus is the largest and most prominent subnuclear organelle, present in nearly all eukaryotic cell types and taking up a large percentage of nuclear volume. Because of its dense protein composition and prominent structure, the nucleolus is readily visible under light microscopy. This led to its first description in somatic cells almost 200 years ago, in the early 1830s (Valentin and Barry, 1836), laying the groundwork for our understanding of this vital organelle.

Following extensive cytological description in many systems (Montgomery, 1898), the nucleolus was found, in the 1960s, to play a crucial role in somatic cells as the coordinator of ribosome biogenesis. The nucleolus provides the site of ribosomal (rRNA) transcription and processing, as well as assembly of processed rRNA with ribosomal proteins (Brown and Gurdon, 1964; Edström et al., 1961; Perry, 1962). In following years, further studies implicated the nucleolus in a plethora of other processes, including DNA repair, cell cycle regulation, stress response and viral infection, defining the nucleolus as ‘pluri-’ or ‘multi-’ functional (Boisvert et al., 2007; Dubois and Boisvert, 2016; Pederson, 1998). Many of the non-ribosomal functions of the nucleolus are related to biogenesis of other ribonucleoprotein complexes, such as telomerase, the signal recognition particle and the spliceosome (Dubois and Boisvert, 2016). More recently, links between nucleolar dysregulation and pathologies such as ageing, cancer, neurodegeneration and developmental disorders have emerged (Corman et al., 2023; Correll et al., 2019; Penzo et al., 2019) (Box 1).

**Box 1. Links between nucleolar chromatin and the role of the nucleolus in aging, cancer and other pathologies**

In the light of our new knowledge about the nucleolus and nucleolar chromatin in development, it will be important in future to explore how these aspects may impact disease or pathology. The nucleolus has long been observed to change in cancer (MacCarty, 1936), and both increasing nucleolar size and nucleolar activity are associated with worse prognosis (Derenzini et al., 1989; Williamson et al., 2006). Additionally, extra-ribosomal functions are already noted for the nucleolus in cancer. This includes stress-mediated p53 regulation (Holmberg Olausson et al., 2012), cell cycle arrest (Sherr, 2006) or genome stability (Lindström et al., 2018). However, although a potential link between NAD organisation and nucleolar heterochromatin in cancer has been hypothesised (Quin et al., 2014), this has not been investigated. Both nucleolar and lamina dysfunction are also implicated in aging and senescence (Kasselimi et al., 2022). Nucleolar activity increases in the premature aging disorder Hutchinson-Gilford progeria syndrome (Buchwalter and Hetzer, 2017), while, conversely, smaller nucleoli are associated with longevity (Tiku et al., 2017). A causative link between Pol I/III activity and longevity regulation has indeed been demonstrated in multiple organisms (Filer et al., 2017; Martínez Corrales et al., 2020). Given the global changes to heterochromatin known to occur in aging and senescence (Booth and Brunet, 2016; Criscione et al., 2016; Shaban and Gasser, 2025), it is tempting to speculate whether nucleolar heterochromatin disruption may also play a role in these pathologies.

Novel tools and concepts (e.g. nucleolar DNA mapping, liquid–liquid phase separation and high-resolution imaging) have led to new understandings of the nucleolus. This Review explores unexpected roles for the nucleolus in mammalian development, with a particular focus on the nucleolus as a regulator of genome organisation. We first provide an overview of the structure and function of the nucleolus. We then outline the reprogramming of nucleoli during mammalian pre-implantation development and describe the roles of nucleolar factors in development, including evidence from mouse models and human clinical conditions. Finally, we discuss the recent advances in understanding nucleolar regulation of chromatin, and outline novel genomics approaches to identifying nucleolar-associated DNA.

## The nucleolus – overview of structure and function

**Fig. 1.**
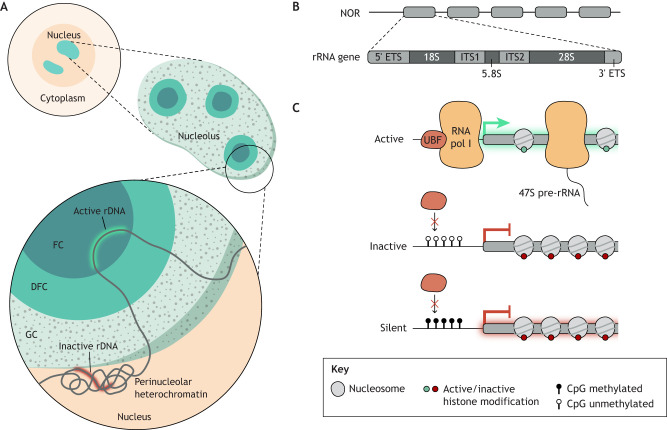
**Nucleolar structure and rRNA gene organisation.** (A) Detailed view of nucleolar architecture. The nucleolus is organised into three distinct regions: the fibrillar centre (FC), the dense fibrillar component (DFC) and the granular component (GC). Surrounding the nucleolus is a shell of heterochromatin, which includes silent ribosomal RNA genes (red). DNA loops into the nucleolus, positioning arrays of active rRNA genes (green) within the FC, where transcription occurs. (B) Schematic of rRNA gene organisation. rRNA genes are arranged in tandem repeats, forming nucleolar organiser regions (NORs). Each rRNA gene contains coding sequences for the 18S, 5.8S and 28S subunits, which are separated by internal transcribed spacers (ITS1 and ITS2) and flanked by external transcribed spacers (ETS). (C) rRNA genes exist in three transcriptional states: active, inactive and silent. Each state is characterised by specific histone modifications, DNA (CpG) methylation patterns and associated transcription factors. Silent rRNA gene promoters are marked by repressive histone modifications, such as H3K9me2/3 and DNA methylation, which prevents binding of the RNA Pol I activator upstream binding factor (UBF). Inactive and active rRNA genes both lack DNA methylation at their promoters. Active rRNA genes are bound by UBF and are therefore actively transcribed. Inactive genes lack UB, but, due to the absence of DNA methylation, they are primed for potential activation upon UBF binding.

The nucleolus is composed of three distinct ultra-structurally defined regions, each dedicated to a specific stage in the sequential process of ribosome synthesis (Dubois and Boisvert, 2016) (Fig. 1A). The nucleolus lacks a surrounding membrane but is compartmentalised within the nucleus through a physicochemical process known as liquid–liquid phase separation (Feric et al., 2016). This unique characteristic allows the nucleolus to maintain its structural integrity and functional specificity (Lafontaine et al., 2021).

The fibrillar centre (FC; abbreviations are summarised in Box 2) serves as the site for ribosomal RNA (rRNA) gene transcription by RNA polymerase I (Pol I), producing a single, long precursor molecule known as the 47S pre-rRNA. This precursor is subsequently processed within the dense fibrillar component (DFC), where it is cleaved into smaller rRNA species: the 18S rRNA, which becomes part of the 40S (small) ribosomal subunit; and the 5.8S and 28S rRNAs, which are integral to the 60S (large) ribosomal subunit. The final stages of ribosome assembly occur in the granular component, which surrounds the FCs. Here, the individual rRNAs are further processed and assembled with ribosomal proteins, resulting in the formation of nearly complete pre-ribosomal subunits. These subunits are then exported to the cytoplasm, where they undergo final maturation and assembly into functional ribosomes (Bohnsack and Bohnsack, 2019; Dörner et al., 2023; Tschochner and Hurt, 2003).

**Box 2. Abbreviations**

2C-like cells – two-cell-like cells

Chr19 – chromosome 19

DamID – DNA adenine methyltransferase identification

DFC – dense fibrillar component

ESC – embryonic stem cell

FC – fibrillar centre

FISH – fluorescent *in situ* hybridisation

ICM – inner cell mass

LAD – lamina-associated domain

lncRNA – long non-coding RNA

LoNA – long nucleolus-specific lncRNA

MEF – mouse embryonic fibroblast

NAD – nucleolar-associated domain

NCC – neural crest cell

NLB – nucleolus-like body

NOR – nucleolar organiser regions

NPBs – nucleolar precursor bodies

NPC – neural progenitor cell

NSN – non-surrounded nucleolus

O-MAP – oligonucleotide-mediated proximity-interactome mapping

RNA Pol I – RNA polymerase I

rDNA – ribosomal DNA

rRNA – ribosomal RNA

SN – surrounded nucleolus

snoRNA – small nucleolar RNA

SPRITE – split-pool recognition of interactions by tag extension

TAD – topologically associated domain

TE – transposable element

TSA-seq – tyramide signal amplification sequencing

UBF – upstream binding factor

Xi – inactive X chromosome

ZGA – zygotic genome activation

The nucleolus forms around chromatin regions containing rRNA genes, which are organised into tandem arrays of 47S-encoding gene repeats separated by long intergenic spacers (Gonzalez and Sylvester, 1995; Sylvester et al., 1986) (Fig. 1B). These arrays are referred to as nucleolar organiser regions (NORs). Multiple copies of these rRNA gene arrays are distributed across several chromosomes, resulting in hundreds to thousands of ribosomal DNA (rDNA) copies per genome (Henderson et al., 1972; Prokopowich et al., 2003). This redundancy is crucial to meet high cellular demand for rRNA molecules.

Despite the abundance of rDNA copies, not all are transcriptionally active. In somatic mammalian cells, rRNA genes are categorised into three groups based on their transcriptional state, each characterised by distinct epigenetic patterns: silent, inactive and active genes (Bersaglieri et al., 2022; Bersaglieri and Santoro, 2019; Birch and Zomerdijk, 2008; Goodfellow and Zomerdijk, 2013) (Fig. 1C). Silent rRNA genes exhibit a tightly packed heterochromatic state and are marked by repressive histone modifications, such as H3K9me2/3. These genes also carry CpG methylation at their promoter regions, which prevents binding of the RNA Pol I activator upstream binding factor (UBF), thereby maintaining transcriptional silencing. Additionally, silent rRNA genes are localised outside of the FC–DFC border regions, closely associated with a shell of heterochromatin surrounding the nucleolus, known as perinucleolar heterochromatin (Akhmanova et al., 2000). This arrangement spatially separates silent copies from the sites of active rDNA transcription.

In contrast, both inactive and active rRNA genes lack DNA methylation at their promoters, distinguishing them from silent rRNA copies. The primary difference between inactive and active genes lies in their association with UBF. Active rRNA genes are bound by UBF, enabling the assembly of the pre-initiation complex and subsequent Pol I-mediated transcription. UBF binding is retained during mitosis, when the nucleolus disassembles, ‘bookmarking’ active rDNA repeats and facilitating nucleolus reassembly at telophase (Grob et al., 2014). Inactive genes, however, are not associated with UBF and remain in a nucleosome-packed chromatin state, which keeps them transcriptionally silent. Nevertheless, the absence of promoter DNA methylation in these inactive genes primes them for potential activation upon UBF binding. Conversely, depletion of UBF can convert active rRNA genes into an inactive state, demonstrating the dynamic regulation of rRNA gene transcription (Birch and Zomerdijk, 2008). Interestingly, in contrast to somatic cells, embryonic stem cell (ESC) nucleoli hypertranscribe rRNA and all rDNA copies are in an active state, reflecting generally open chromatin and a high requirement for biosynthesis. Indeed, increased ribogenesis can be considered a general feature of stem cell biology (reviewed by Saba et al., 2021). Upon differentiation of ESCs to somatic lineages, a subset of rDNA loci are rendered silent by the establishment of heterochromatin (Guetg et al., 2010; Mayer et al., 2006; Santoro et al., 2010; Savic et al., 2014).

## Nucleoli in development and developmental disorders

The complex tripartite structure of active nucleoli is typical of most somatic cell types. However, nucleoli in early cleavage stage embryos are initially found in an atypical ‘immature’ state, termed nucleolar precursor bodies (NPBs), which undergo dramatic remodelling to form mature somatic-like nucleoli as embryogenesis proceeds (reviewed by Kresoja-Rakic and Santoro, 2019). In this section, we discuss the importance of the nucleolus in development, both during nucleolar reprogramming in preimplantation embryos, and the evidence from mouse and human suggesting that nucleolar factors underlie cell type-specific developmental phenotypes.

### Nucleolar reprogramming in the early mouse embryo and a link with totipotency

**Fig. 2.**
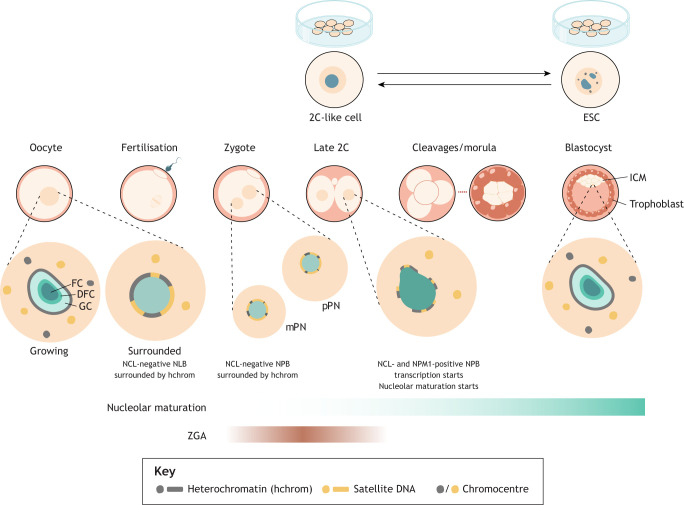
**Maturation of the nucleolus and chromatin during mouse preimplantation development.** In growing oocytes, nucleoli exhibit a tripartite structure and are surrounded by heterochromatin. Upon completion of oocyte growth, two types of nuclear organisation emerge: oocytes with a non-surrounded nucleolus (NSN, not shown) and surrounded nucleolus (SN). In SN oocytes, the nucleolus ceases transcription and is termed a nucleolus-like body (NLB). SN oocytes lack chromocentres, and satellite DNA relocates to perinucleolar heterochromatin. At meiosis, NLBs disappear, reforming in the zygote pronuclei (mPN, maternal; pPN, paternal) as nucleolar precursor bodies (NPBs). NPBs lack somatic nucleolar protein such as nucleolin (NCL) and are surrounded by DNA primarily composed of satellite DNA. After zygotic genome activation (ZGA), nucleolar and chromatin maturation begins. By the late two-cell (2C) stage, the NPB starts to accumulate nucleolar proteins, including NCL and nucleophosmin 1 (NPM1). rRNA transcription initiates at the NPB periphery, heterochromatin assembles around the NPB and chromocentres start to form. With subsequent cleavages, the nucleolus continues to mature. By the 16-cell stage, the nucleolus has a somatic-like tripartite structure. Two *in vitro* models (top) represent different developmental stages: two-cell-like (2C-like) cells display features of the 2C embryo; and embryonic stem cells (ESCs) represent the pluripotent epiblast. DFC, dense fibrillar component; FC, fibrillar centre; GC, granular component; ICM, inner cell mass.

The period of pre-implantation development following fertilisation of the egg by a sperm is characterised by drastic reprogramming of the chromatin states of both parental genomes. DNA methylation, histone modifications and genome organisation are all dynamically reset during this window (Du et al., 2022). Chromatin reprogramming is thought to be necessary for establishing totipotency: the state characteristic of the one- to two-cell stage in mice, whereby a single cell can give rise to all embryonic and extra-embryonic cell lineages (Genet and Torres-Padilla, 2020). During the period of chromatin remodelling, an intriguing reprogramming of the nucleolus and its associated chromatin also occurs; this process has been best characterised in the mouse (Fig. 2).

During oocyte growth, the nucleolus displays a canonical tripartite structure and actively transcribes rRNA. In growing oocytes, the chromatin is dispersed throughout the nucleoplasm and does not encircle the nucleolus. As the oocyte reaches maturity at the fully grown oocyte stage, the nucleolus forms a large spherical structure, termed the nucleolus-like body (NLB) (Chouinard, 1971). The NLB contains many nucleolar proteins, as well as RNA, yet it is depleted of rRNA (Fulka and Langerova, 2014; Shishova et al., 2015). In maturing oocytes, transcription ceases and the nucleolus becomes inactive. Concomitantly, chromatin rearranges to form a shell surrounding the inactive NLB (surrounded nucleolus, SN). Not all fully grown oocytes adopt this chromatin configuration, however; some remain in a non-surrounded nucleolus (NSN) state (Bouniol-Baly et al., 1999; Debey et al., 1993; Kopečný et al., 1995; Zuccotti et al., 1995). Upon ovulation and progression to metaphase II, the NLB dissolves. Interestingly, while both NSN and SN oocytes can be ovulated and subsequently fertilised, only SN oocytes are developmentally competent: they have higher rates of meiotic maturation and development to blastocyst, whereas fertilised NSN oocytes arrest at the two-cell stage (Liu and Aoki, 2002; Zuccotti et al., 1998, 2002).

Following fertilisation, NPBs are formed and persist until the early to mid two-cell embryo stage (Flechon and Kopecny, 1998). NPBs lack rRNA transcription and a tripartite structure, and are conspicuously encircled by a ring of DNA (Martin et al., 2006; Zatsepina et al., 2003). RNA fluorescent *in situ* hybridisation (FISH) experiments have defined that this ring comprises – at least partially – pericentric major satellite and centromeric repeat sequences, in addition to some rDNA loci (Aguirre-Lavin et al., 2012). From the late two-cell stage, a gradual transformation towards mature somatic-type nucleoli ensues. Major satellite sequences (and potentially other NPB-associated DNA) move away from the NPB to the newly forming chromocentres, the canonical sites of constitutive heterochromatin in mouse cells (Martin et al., 2006). At this time, rRNA transcription is initiated (Zatsepina et al., 2003) and, by the 16-cell stage, nucleoli fully adopt a complex, somatic-type structure (Flechon and Kopecny, 1998) (Fig. 2).

In the mouse, the initiation of transcription from the genome of the embryo, known as zygotic genome activation (ZGA), begins from the one-cell stage, peaking at the two-cell stage (Aoki et al., 1997; Hamatani et al., 2004; Sakamoto et al., 2024). Immature nucleoli in the form of NPBs are, therefore, present at totipotency and ZGA, and the onset of nucleolar maturation correlates with the transition from totipotency to pluripotency as embryonic development progresses (Fig. 2). ZGA may indeed drive nucleolar maturation by initiating rRNA hypertranscription, which in turn supports nucleolar structure (Dash et al., 2023; Xie et al., 2022).

Totipotency and pluripotency can be modelled *in vitro*, by comparing two-cell-like cells (2C-like cells) and ESCs. 2C-like cells are a rare, spontaneously emerging sub-population of ESC cultures that show features similar to the two-cell embryo, including expression of MERVL (murine endogenous retrovirus-L) transposable elements (TEs) and ZGA genes (Bošković et al., 2014; Genet and Torres-Padilla, 2020; Ishiuchi et al., 2015; Macfarlan et al., 2012). Using the 2C-like model system, several recent studies have uncovered a link between nucleolar remodelling and totipotency. Nucleolar morphology of 2C-like cells mimics NPBs, appearing smooth and rounded in comparison with multi-lobed ESC nucleoli (Percharde et al., 2018; Xie et al., 2022; Zhu et al., 2021) (Fig. 2). 2C-like cell nucleoli are also less active, displaying dampened rRNA synthesis, similar to NPBs (Xie et al., 2022). Intriguingly, disruption of nucleoli using inhibitors of RNA Pol I is sufficient to induce a 2C-like state in a high proportion of ESCs, demonstrating a link between nucleolar structure and function, and totipotency-like characteristics (Xie et al., 2022; Yu et al., 2021). In a similar finding, ESC cultures forced to remain in the G1 phase, where nucleoli are less mature, also show an increase in 2C-like cells (Zhu et al., 2021). *In vivo*, the inhibition of nucleolar maturation prevents mouse embryos from developing beyond the two-cell stage (Li et al., 2023; Xie et al., 2022; Yu et al., 2021). Additional evidence linking nucleolar reprogramming to totipotency comes from somatic cell nuclear transfer embryos, in which development rates are typically low (Gouveia et al., 2020). Treatment of donor cells with mTOR inhibitors or inhibitors of RNA Pol I enhances the reversal of somatic-nucleoli to NPBs, and improves rates of development to blastocyst in somatic cell nuclear transfer embryos (Liao et al., 2020). These studies suggest a close link between nucleolar maturation and the totipotency to pluripotency transition *in vivo*, and between the 2C-like to ESC transition *in vitro*. However, treatment with mTOR or RNA Pol I inhibitors may also impact mRNA translation independently of altered nucleolar structure, making it difficult to unequivocally define the importance of nucleolar morphology. Intriguingly, however, the proposed mechanisms behind such a link hinge upon changes to nucleolar chromatin and the orchestration of genome organisation, an idea explored further below.

### Mouse models of nucleolar dysfunction in development

The particular importance of the nucleolus in development is underscored by the early phenotypes of mouse models of nucleolar factors (Table 1). Loss of key proteins involved in rRNA synthesis exhibit early pre- or post-implantation lethality, revealing a requirement for high nucleolar function at these stages. For example, loss of the histone chaperone *Hira* disrupts rRNA synthesis as early as the one- to two-cell stage (Lin et al., 2014). Absence of crucial nucleolar proteins, such as RNA Pol I (*Polr1a*) or fibrillarin (*Fbl*), causes morula arrest (Falcon et al., 2022; Newton et al., 2003). Furthermore, the disruption of other factors, such as NPM1 or nucleolin, manifests as developmental arrest around mid-gestation (Doron-Mandel et al., 2021; Grisendi et al., 2005). The reason for the varying severity upon loss of different factors is unknown. One possibility is a variation in the degree of rRNA reduction in each model. Some factors may also carry out non-redundant, independent functions, as well as promoting rRNA synthesis. For example, *Fbl* depletion also induces loss of the small nucleolar RNA (snoRNA) U76, which is potentially involved in rRNA modification (Newton et al., 2003). Alternatively, maternally provided mRNAs for key nucleolar proteins may compensate for and delay lethality in zygotic knockouts.

**Table 1.** Example mouse models of nucleolar dysfunction

Gene	Mutation	Defect	Nucleolar-related mechanism	Stage when defect is apparent	Initial reference
Nucleolin (*Ncl*)	*Ncl^ΔGAR/ΔGAR^*	Lethality	Unknown	<E10.5	Doron-Mandel et al. (2021)
Fibrillarin (*Fbl*)	*Fbl^GT/GT^*	Embryo arrest and apoptosis	Unknown and snoRNA synthesis	Morula	Newton et al. (2003)
*Chd1*	*Chd1^−/−^ Chd1^f/f^* and *Sox2-Cre*	Lethality	Hypotranscription and reduced rRNA synthesis	E9.5	Guzman-Ayala et al. (2015)
	*Chd1^f/f^* and *Tie2-Cre*	Loss of HSPCs and lethality	Hypotranscription and reduced rRNA synthesis	E15.5	Koh et al. (2015)
*Npm2*	*Npm2^−/−^* (M-KO)	Embryo arrest	Absence of NPBs	Two cell	Burns et al. (2003)
*Hira*	*Hira^f/f^* and *Zp3-cre* (M-KO)	Embryo arrest	Reduced rRNA synthesis	Zygote	Lin et al. (2014)
Nucleophosmin (*Npm1*, *B23*)	*Npm1^−/−^*	Lethality and defective haematopoiesis	Genomic instability and defective ribogenesis	E11.5-E12.5	Grisendi et al. (2005)
*Polr1a*, *Polr1c* or *Polr1d*	*Polr1a^−/−^ Polr1c^−/−^ Polr1d^−/−^*	Embryo arrest	Reduced rRNA synthesis	Morula	Falcon et al. (2022); Smallwood et al. (2023); Chen et al. (2008)
*Polr1b*	*Rpo1-2(Polr1b)^−/−^*	Embryo arrest	Reduced rRNA synthesis	Morula	Falcon et al. (2022); Smallwood et al. (2023); Chen et al. (2008)
*Tcof1*	*Tcof1^+/−^*	Neonatal lethality and craniofacial abnormalities	Reduced rRNA, apoptosis and neural crest hypoplasia	P0	Dixon et al. (2006)
*Ubtf*	*Ubtf^Δ/Δ^*	Embryo arrest	Failure to form pre-initiation complex and reduced rRNA	Morula	Hamdane et al. (2014)

E, embryonic day; HSPCs, hematopoietic stem and progenitor cells; M-KO, maternal knockout; NPBs, nucleolar precursor bodies; P, postnatal day; rRNA, ribosomal RNA; snoRNA, small nucleolar RNA.

Mouse loss-of-function models that progress beyond gastrulation have frequent tissue-specific developmental effects. For example, defects in haematopoiesis are frequently documented upon defective ribogenesis (Grisendi et al., 2005; Koh et al., 2015), despite widespread expression of nucleolar factors across multiple tissues and not only blood lineages. This is supported by experiments in which RNA Pol I inhibition in *ex vivo* culture reduced the formation of hematopoietic stem cells (Liu et al., 2024). Similarly, the expression of RNA Pol I subunits and the nucleolar protein TCOF1 is upregulated in the developing neural crest; mutations in these factors drive craniofacial defects and neonatal lethality (Dixon et al., 2006; Falcon et al., 2022; Smallwood et al., 2023). These findings underscore the importance of mechanisms to drive hypertranscription and increase ribogenesis in certain progenitor populations to support their expansion (Percharde et al., 2017). It will be important in future studies to investigate how distinct cells and tissues precisely coordinate rRNA levels and proliferation.

Interestingly, embryonic phenotypes produced upon depletion of nucleolar factors are not always due to defective rRNA synthesis. These data point to additional unexplored functions of the nucleolus in development. Enucleolation experiments, in which the NLB is removed from oocytes by micromanipulation and NPBs subsequently fail to form after fertilisation, have revealed that the maternal NLB is essential for progression beyond the two-cell stage, even in the absence of rRNA expression defects (Fulka and Langerova, 2014; Kyogoku et al., 2014; Ogushi et al., 2008). Maternal deletion of the major NPB protein NPM2 phenocopies loss of NPBs, and NPM2 overexpression is sufficient to rescue embryogenesis after enucleolation (Burns et al., 2003; Ogushi et al., 2017). Defects upon NPM2 and/or NPB absence include incomplete sperm decondensation, higher-order chromatin disorganisation and chromosome segregation defects (Inoue et al., 2011; Ogushi and Saitou, 2010; Ogushi et al., 2017). Despite this, the exact role of the NPB and mechanisms of chromatin organisation in these early stages is still unclear.

### The regulation and role of nucleolar reprogramming in human development

While much of our knowledge of nucleolar reprogramming relies on model organisms, particularly the mouse (Fig. 2), available evidence suggests similar processes are important during human development. Observations of possible nucleolar maturation in human embryos were made via electron microscopy as early as 1986 (Tesařík et al., 1986, 1987). These studies suggested that, in two-to four-cell stage human embryos, nucleoli exist as spherical, unstructured NPBs surrounded by chromatin, and progress to form mature nucleoli by the morula stage. The first signs of possible nucleolar maturation were observed at the six- to eight-cell stage, when chromatin first infiltrates the NPBs, followed by the onset of rRNA synthesis and an increase in nucleolar granularity (Tesařík et al., 1987, 1986). Initiation of nucleolar maturation at the six- to eight-cell stage, concomitant with human ZGA, suggests that these processes may be linked. The mechanisms underlying the ultrastructural changes in nucleoli of human embryos remain to be explored. In the future, modern molecular techniques applied to human samples will start to reveal answers to these questions. Highlighting the importance of nucleolar maturation and function in human development, several variants in nucleolar factors are known to underlie human developmental differences, as described below.

### Nucleolar pathologies drive developmental differences

Although all cells require rRNA synthesis, there are several tissue-specific developmental differences due to variants in nucleolar proteins, termed ribosomopathies (reviewed by Mills and Green, 2017; Ni and Buszczak, 2023; Yelick and Trainor, 2015). Variants in the genes encoding RNA Pol I subunits or the co-factor TCOF1 give rise to neurological, cardiac, craniofacial and/or limb developmental differences (Dauwerse et al., 2011; Misceo et al., 2023; Smallwood et al., 2023; Sulik et al., 1987; Weaver et al., 2015; Yelick and Trainor, 2015). Experiments so far in mouse and human suggest that these conditions are caused by defective ribogenesis in neural crest cells (NCCs), which express particularly high levels of RNA Pol I subunits (Falcon et al., 2022; Smallwood et al., 2023). Missense variants in *UBTF* (encoding UBF) also result in childhood-onset neurodegeneration with brain atrophy (CONDBA) (Edvardson et al., 2017; Tinker et al., 2022; Toro et al., 2018), which causes neuro-regression from very early in life. Interestingly, this condition is linked to rRNA overproduction (Edvardson et al., 2017), highlighting the importance of precise nucleolar homeostasis.

As well as defects in the genes responsible for ribogenesis, variants in factors unrelated to rRNA production can also cause nucleolar defects and developmental differences. First shown with repeat expansion of the gene *C9orf72*, which causes amyotrophic lateral sclerosis, basic peptides can cause protein aggregation in nucleoli and cell death (Kwon et al., 2014). Recently, this mechanism has been suggested to occur for many variants associated with developmental conditions, with the generation of poly-arginine repeats within variant proteins sufficient to cause nucleolar mis-partitioning and disruption of nucleolar function. For example, HMGB1 mis-partitioning to the nucleolus is associated with the complex malformation syndrome BPTAS (brachyphalangy-polydactyly-tibial aplasia/hypoplasia syndrome). In contrast, other variants in HMGB1 that do not cause localisation to the nucleolus are associated with distinct phenotypes (Mensah et al., 2023).

An interesting question is why such conditions affect only certain tissues or organs, given the often-ubiquitous expression of factors involved in ribogenesis. One possibility mentioned above is the increased energetic demand of certain cell types, such as NCCs (Falcon et al., 2022; Smallwood et al., 2023), making them particularly sensitive to nucleolar disruption. Alternatively, some cell types might be more susceptible to nucleolar stress upon rRNA downregulation. NCCs possess increased sensitivity to rDNA damage and P53 activation, which occurs upon nucleolar stress (Calo et al., 2018). Indeed, the chromatin remodeller CHD1 is required for the repair of GC-rich genes and rDNA, which accumulate hypertranscription-associated DNA damage in early post-implantation embryos (Bulut-Karslioglu et al., 2021). Future work is needed to investigate these and other possibilities, as well as to investigate other mechanisms linking nucleolar dysfunction to pathology.

## Approaches to study nucleolar-associated DNA and genome organisation

An emerging role for the nucleolus in development relates not to ribogenesis but to DNA organisation around the nucleolar periphery. DNA within the interphase nucleus is arranged in a non-random 3D configuration, with each individual chromosome occupying a distinct territory (Cremer and Cremer, 2010). The radial position of DNA regions is influenced by genomic characteristics such as gene density and chromosome size (Boyle et al., 2001; Sun et al., 2000). For example, highly transcribed euchromatic regions are typically localised towards the interior of the nucleus, while heterochromatin is often localised in DNA regions associated with the nuclear lamina called lamina-associated domains (LADs) (Guelen et al., 2008; Peric-Hupkes et al., 2010). Similarly, the chromatin surrounding nucleoli can be mapped to identify nucleolar-associated domains (NADs) (Table 2). NADs comprised NORs, as well as non-rDNA containing regions, and are typically heterochromatic, similar to LADs.

**Table 2.** Methodologies for identifying nucleolar-associated DNA

Method type	Method	References
Physical isolation of nucleoli	Nucleolar fractionation and DNA-seq	Nemeth et al. (2010); van Koningsbruggen et al. (2010); Dillinger et al. (2017); Vertii et al. (2019); Bizhanova et al. (2020)
	NoLMseq	Walavalkar et al. (2024 preprint)
Proximity labelling by protein or RNA target	Nucleolar DamID	Bersaglieri et al. (2022)
	RNA O-MAP	Tsue et al. (2024)
	TSA-seq	Chen et al. (2018); Kumar et al. (2024)
3D genome interactions	rDNA Hi-C	Yu and Lemos (2018); Bersaglieri et al. (2022)
	Nucleolar Hi-C*	Peng et al. (2023)
	SPRITE	Quinodoz et al. (2018)

*Nucleolar Hi-C combines physical isolation of nucleoli and 3D genome methods.

DamID, DNA adenine methyltransferase identification; NoLMseq, nucleolar laser microdissection sequencing; rDNA, ribosomal DNA; RNA O-MAP, oligonucleotide-mediated proximity-interactome MAPping; SPRITE, split-pool recognition of interactions by tag extension; TSA-seq, tyramide signal amplification sequencing.

### A burst of innovative genome-wide mapping techniques has identified NADs in many cellular contexts

Genome-wide, base-level identification of the DNA loci associated with nucleoli was first achieved by biochemical isolation of nucleoli using sonication and sucrose gradient fractionation, followed by DNA sequencing (Dillinger et al., 2017; Nemeth et al., 2010; van Koningsbruggen et al., 2010). Subsequently, a growing number of techniques have been developed to permit identification of NADs (Table 2). Initial studies found that NADs make up between 4% and 38% of the genome, and highlighted that NADs are predominantly heterochromatic, and frequently overlap with regions annotated as LADs (Dillinger et al., 2017; Nemeth et al., 2010). Regions identified as NADs include centromeric and pericentromeric repeats, lowly transcribed regions, and clustered gene families, such as zinc finger genes and olfactory receptors (Nemeth et al., 2010; van Koningsbruggen et al., 2010).

Subsequently, other approaches have been developed to identify NADs. The chromosome conformation capture method Hi-C has been adapted to identify DNA regions interacting with nucleoli, either by biochemical isolation of nucleoli before library generation [i.e. nucleolus-enriched Hi-C (Peng et al., 2023)] or by specifically interrogating DNA regions that interact with rDNA (Bersaglieri et al., 2022; Yu and Lemos, 2018). Split-pool recognition of interactions by tag extension (SPRITE) captures all-by-all 3D genome DNA interactions by crosslinking nuclei and, through repeated rounds of splitting and tagging, labels interacting molecules with unique barcode combinations (Quinodoz et al., 2018). Upon sequencing, sequences sharing barcodes are reconstructed to identify interacting regions. SPRITE performed in mouse ESCs has identified multi-locus interactions of inactive regions containing rDNA regions, termed inactive hubs. The SPRITE protocol also captures RNA-DNA interactions, and rRNA is enriched at loci in the inactive hub. In contrast, active hubs are associated with nuclear speckles.

Recent techniques have used variations of proximity labelling to tag nucleolar DNA, avoiding difficulties associated with fractionation and biochemical purification. One such example is nucleolar DamID, in which expression of a nucleolus-directed histone-Dam fusion labels NADs with adenine methylation. Enrichment of adenine methylation can then be read using bisulfite sequencing to identify NADs (Bersaglieri et al., 2022). A recently developed method, oligonucleotide-mediated proximity-interactome mapping (O-MAP), can detect RNA, protein or DNA molecules interacting with particular RNAs *in situ* (Tsue et al., 2024). By first annealing a FISH probe specific to the RNA of interest, followed by a horseradish peroxidase-conjugated secondary probe, proteins near the RNA of interest can be biotinylated. Pulldown of biotinylated material from cross-linked cells can thus identify nucleic acids or proteins in proximity to the RNA of interest through sequencing or mass spectrometry. Using primary probes against 47S pre-rRNA, the authors successfully identified NADs in HeLa cells and pancreatic ductal adenocarcinoma cell lines. Similarly, tyramide signal amplification sequencing (TSA-seq) has recently been applied to the detection of NADs (Chen et al., 2018; Kumar et al., 2024). TSA-seq couples horseradish peroxidase to an antibody targeting a protein of interest, generating a gradient of free biotinyl-tyramide, which labels surrounding molecules as a function of their distance from the target protein. Labelled material is pulled down and the DNA is sequenced to detect interacting loci. TSA-seq, therefore, differs slightly from DamID and O-MAP in that the signal is a molecular ruler, decaying with distance from the target, as opposed to labelling within a proximity radius. Finally, a recent preprint describes NoLMseq, where nucleoli are physically isolated from individual cells by laser-capture microdissection, before isolating and amplifying DNA by whole-genome amplification for sequencing (Walavalkar et al., 2024 preprint). NoLMseq and single-cell SPRITE are notably the first techniques to identify NADs in single cells.

**Fig. 3.**
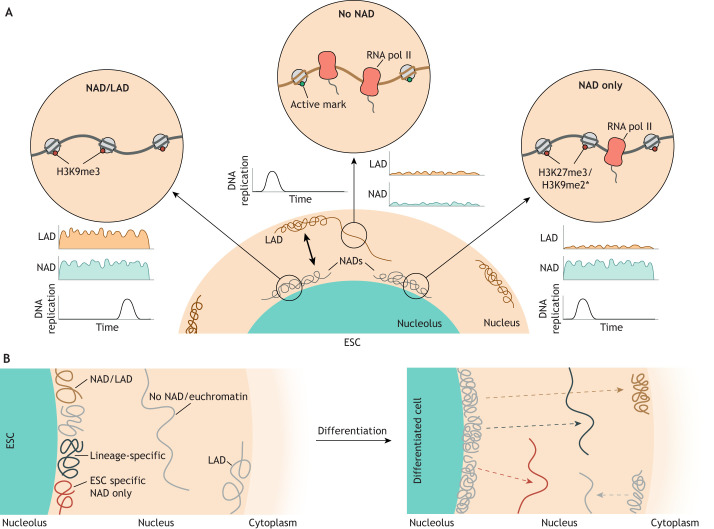
**Features of nucleolar-associated domains in different cell types.** (A) Annotation of genomic regions into different nucleolar-associated domain (NAD) types. NADs can be divided into regions that are found only in NADs (NAD only, also called type II) and regions that are also found in lamina-associated domains (NAD/LAD, also called type I). Type I are enriched in H3K9me3, display very low gene density and gene expression, and are late replicating. Type II are early replicating and enriched for facultative heterochromatin. Depending on the study, type II are enriched for H3K9me2 or H3K27me3 (asterisk). Those regions that are not found enriched at nucleoli in NAD-mapping assays can be defined as no NAD. No NADs tend to be actively transcribed regions, decorated with active epigenetic marks and early replicating. (B) Changes in NADs during differentiation of embryonic stem cells (ESCs). NADs become more rigid and stable upon differentiation. A subset of ESC NADs changes location after differentiation, repositioning to the nucleoplasm or to LADs in a manner seemingly instructed by their status as type I or type II in ESCs. Loci that reposition to the nucleoplasm are enriched for lineage-specific and developmental genes (Bersaglieri et al., 2022; Bizhanova et al., 2020).

NAD sequences identified in different studies have followed similar trends (NADs are largely found as heterochromatic regions with low gene expression). Within a given cell type, two subsets of NADs can be identified based on genomic characteristics (Fig. 3A). NAD/LAD sequences (also called type I) make up a larger fraction of the genome, overlap with LADs, and are gene poor, enriched in constitutive heterochromatin marks (e.g. H3K9me3) and are late replicating (Bizhanova et al., 2020; Vertii et al., 2019). NAD/LAD are thought to represent regions that can be located at either the nucleolus or the nuclear lamina in individual cells of a population. In contrast, NAD only sequences (also called type II) make up smaller fraction of the genome, do not overlap with LADs and are enriched for facultative heterochromatin marks (e.g. H3K27me3) (Vertii et al., 2019).

The NADs identified in different studies also vary significantly, indicating cell type-specific NAD establishment during development and differentiation. However, the variety of techniques used across different studies means that some of the variation in NAD sequence characteristics between cell types may also be driven by technical differences in methodology. For example, NADs identified by SPRITE and TSA-seq do not often overlap with LAD regions, in contrast to other techniques (Kumar et al., 2024; Quinodoz et al., 2018). Nucleolar fractionation via sonication is likely to skew the recovered NAD sequences strongly towards heterochromatin, because heterochromatin is sonication resistant (Bersaglieri and Santoro, 2023), which may explain some discrepancies between methods. Similarly, TSA-seq detects the frequency of DNA localising in linear proximity to the nucleolus, as opposed to other methods such as DamID and NAD-seq, which rely on direct molecular contact (Kumar et al., 2024). Understanding the differences between techniques is essential to probe the biological meaning of NAD dynamics. It is also important to consider that several studies have identified NADs in immortalised or cancer cell lines, which may not be indicative of the *in vivo* context. With this in mind, it will be exciting in future to direct these protocols towards achieving a greater understanding of NAD dynamics in stem cells and embryos, as has been recently performed for LADs (Borsos et al., 2019; Guerreiro et al., 2024).

### Additional layers of genomic organisation – the nucleolus also organises genome compartments and TEs

The preferable association of some areas of the genome into NADs and/or LADs has implications for higher-order 3D chromatin organisation. Through approaches such as Hi-C, we now understand genomes to be organised into two compartments: A and B. A compartments are more open and permissive to transcription, whereas B compartments are generally more repressive and heterochromatic (Lieberman-Aiden et al., 2009), and are associated with the lamina or nucleolus (Buchwalter et al., 2019). Such compartments are further divided into topologically associated domains (TADs), regions that show preferential self-interaction and regulation. rDNA repeats fall into repressed chromatin and are enriched for binding of the structural chromatin protein CTCF, suggesting that rDNA may play a role in higher-order genome organisation (Yu and Lemos, 2018). In agreement with this idea, nucleolar Hi-C, which enriches for 3D interactions that include nucleolar-associated DNA, identified 264 such Hi-C NADs in HeLa cells (Peng et al., 2023). Nucleolar disassembly by actinomycin D affected global genome organisation by decreasing compartment strength (Peng et al., 2023). Actinomycin D treatment also caused a breakdown in insulation between TADs, particularly those that were nucleolar adjacent (Peng et al., 2023). In contrast, a separate study has shown that 3D genome organisation remains largely similar in mouse ESCs upon RNA Pol I degradation (Jiang et al., 2020). Such discrepancies may be due to the degree of nucleolar disruption attained in different studies, as well as possible cell type-specific roles for NADs in genome organisation. It will be interesting in future studies to probe in depth the contribution of NAD organisation and rDNA sequences to 3D chromatin structure across developmental stages or tissue types.

Interestingly, a link between the nucleolus and repeat sequences, including TEs, is also emerging in development. A microsatellite repeat locus, *Dxz4*, contains the long non-coding RNA (lncRNA) *Firre*, which is essential for tethering the inactive X chromosome (Xi) to perinucleolar heterochromatin in mouse cells. *Firre* knockdown in mouse embryonic fibroblasts (MEFs) decreases Xi anchoring at the nucleolus and loss of the repressive mark H3K27me3 (Yang et al., 2015). Similarly, *Xist* deletion in mouse cells abrogates Xi targeting to the nucleolus and partial X re-activation (Zhang et al., 2007). LINE1-derived RNA is also crucial for the association of the nucleolar protein nucleolin, with the genomic loci of the ZGA transcription factor *Dux*. *Dux* DNA loci are recruited to perinucleolar heterochromatin in mouse ESCs and embryos for their repression after ZGA (Percharde et al., 2018). The relationship between TEs and the nucleolus extends beyond *Dux*, with genome-wide profiling of LINE1 RNA revealing an enrichment of both its RNA and DNA at the nucleolus. LINE1 enrichment overlaps with NADs, and its depletion induces de-repression of NAD genes in mouse ESCs (Lu et al., 2020). Surprisingly, despite low sequence homology between LINE1 elements in mouse and human, human ESCs rely on LINE1 RNA for gene regulation and nucleolar function (Ataei et al., 2024; Zhang et al., 2024). Together, these findings suggest that the highly repetitive nature of TEs, potential ability of their transcripts to direct phase separation (Lu et al., 2021) and their compartmentalisation in 3D nuclear space may allow them to play conserved roles in nucleolar biology.

### Outlook on overcoming technological limitations to identify NADs in rare cell types

Most of the existing methods for the identification of NADs rely on the availability of abundant input material, and result in the identification of a population-average NAD profile. A technical challenge, therefore, remains to be overcome before the identification of NADs in rare cell populations, pre-implantation embryos or single cells can be achieved. Promisingly, however, several of the methods described above may be amenable to low-input sample types.

Single cell versions of both DamID and SPRITE methods have been developed (Arrastia et al., 2022; Kind et al., 2015). scDamID has been used to detect LADs at single cell resolution, and modification of this technique to target nucleoli (Bersaglieri et al., 2022) may permit identification of NADs in low-input samples. scSPRITE can detect NOR-NOR interactions in single ESCs, but has not yet been demonstrated to identify non-NOR NAD regions in single cells. However, both methods rely on analysis of hundreds of single cells in pseudo-bulk, meaning that these techniques may not be appropriate for very rare sample types. NoLMseq uses laser-capture microdissection to isolate nucleoli before sequencing the associated DNA of individual cells. In this case, the authors used NAD maps from only 53 ESCs to identify two cell populations that differ in their NAD profiles. This method is, therefore, very promising for identification of NADs in rare cell types. One caveat is that the cell type of interest must be amenable to mounting for laser-capture microdissection. Finally, the recently developed expansion *in situ* genome sequencing enables super-resolution detection of nuclear proteins alongside simultaneous DNA sequencing in single cells (Labade et al., 2024 preprint).

## Dynamics and functions of nucleolar chromatin

The earliest examples of nucleolar chromatin reprogramming during mouse development are the transition from NSN to SN in fully grown oocytes and the movement of pericentric major satellites from NPBs to chromocentres in the pre-implantation embryo. Although first observed using microscopy-based techniques, recent NAD-mapping techniques have excitingly suggested that NADs may continue to change throughout development.

Developmental dynamics of NADs have first been identified genome-wide in mouse ESCs compared to more differentiated cell types, such as MEFs, by DNA sequencing of biochemically purified nucleoli (Bizhanova et al., 2020; Vertii et al., 2019) (Fig. 3). The proportion of the genome located in total NADs (incorporating NAD/LAD and NAD only) is higher in MEFs (41%) than in ESCs (31%), and MEF NADs are significantly more enriched in H3K27me3, suggesting that the formation of facultative heterochromatin during differentiation expands the NAD population (Bizhanova et al., 2020). Eighty percent of NADs are shared between ESCs and MEFs. The remaining 20% are, therefore, cell type-specific NADs, and are enriched for developmentally regulated genes, suggesting that variable nucleolar positioning of loci could be one mechanism for lineage-specific gene regulation.

NAD dynamics have also been studied using the paradigm of ESC differentiation to neural progenitor cells (NPCs), in this case using the nucleolar DamID and rDNA Hi-C techniques (Bersaglieri et al., 2022). In this study, NADs have also been found to be gene poor, with low expression levels compared to the genome as a whole. Using Hi-C, changes in 3D genome associations of NADs between cell types have also been found. In ESCs, more varied regions contact the nucleolus but these contacts occur less stably than in NPCs, in keeping with previous findings showing ESC chromatin is more open and dynamic. In contrast to the comparison of ESCs and MEFs (Bizhanova et al., 2020), DamID-NADs are generally depleted of H3K27me3, although differences in H3K9me2/3 have still been seen between distinct NAD subtypes (Fig. 3A). It is unclear if such differences in NAD dynamics are technical or instead reflect the distinct differentiation status of the cell types analysed. Tracking the fate of NADs seen specifically in ESCs has revealed that NAD-only regions cease to be nucleolar associated upon differentiation, moving to the nucleoplasm in NPCs (Fig. 3B). In contrast, NAD/LAD regions unique to ESCs relocated to LADs after differentiation (Bersaglieri et al., 2022) (Fig. 3B). ESC NADs that are released from the nucleolus upon differentiation to NPCs are enriched for neural differentiation-related ontologies; however, these genes were not yet expressed in NPCs. These findings suggest that NAD re-positioning may be a mechanism to either prime genes for activation later in development or to position them at LADs for their ongoing repression.

### Functions of nucleolar chromatin

A crucial function of nucleolar heterochromatin is to silence a subgroup of rDNA repeats. Processed transcripts derived from a subset of intergenic spacers, termed pRNAs, associate with the BAZ2A (also known as TIP5) subunit of the nucleolar remodelling complex to induce the formation of nucleolar heterochromatin and silencing of rDNA (Mayer et al., 2006; Santoro et al., 2010). This mechanism is developmentally regulated: the rDNA repeats lack silencing in ESCs, due to lack of processing of pRNA. pRNA maturation and nucleolar heterochromatin establishment accompanies ESC differentiation and is necessary for exit from pluripotency (Savic et al., 2014).

Beyond its role in regulation of rDNA, what other functions might be performed by nucleolar chromatin? In addition to its role at rDNA loci, TIP5 is also important for the formation of pericentric heterochromatin at major and minor satellite repeats (Guetg et al., 2010). In TIP5-depleted NIH3T3 cells, the number of DAPI-bright pericentric heterochromatin foci and their association with the nucleolus decreased, demonstrating that TIP5 regulation extends beyond rDNA heterochromatin (Guetg et al., 2010).

During development, the NPB of totipotent one- and two-cell stage embryos is also linked to the regulation of pericentric heterochromatin (Fulka and Langerova, 2019). Major satellites and centromeres are frequently located around the NPBs at this stage (Aguirre-Lavin et al., 2012; Probst et al., 2007), and embryos lacking NPBs display disorganised chromatin structure and fail to develop (Ogushi et al., 2017). Mechanistically, transcription of the pericentric major satellite seems to be involved in regulation of heterochromatin reprogramming. Depletion of major satellite transcripts prevents the formation of chromocentres and blocks developmental progression beyond the two-cell stage (Casanova et al., 2013; Probst et al., 2010). Another study has found that major satellite RNAs are involved in reprogramming H3K9me3 to a non-canonical active state present during totipotency, via interaction with SUV39H2 (Burton et al., 2020). In embryos lacking NPBs, the expression of major satellite repeats is reduced at the two-cell stage, suggesting the physical localisation of pericentric heterochromatin around the NPB is important (Fulka and Langerova, 2014). Additionally, the expression of major satellites is reduced in embryos treated with inhibitors of RNA Pol I. Foci of rRNA and major satellite transcription frequently colocalise, suggesting that expression of rRNA might promote an environment permissive to major satellite expression (Chebrout et al., 2022).

While potentially activating roles for NPB chromatin have been reported above, the perinucleolar chromatin of maturing nucleoli is important for gene repression. The 2C-like to ESC transition is accompanied by increased nucleolar chromatin that serves to repress *Dux* expression. More broadly, genes in type II NADs also increase in expression upon nucleolar disruption in ESCs. These data suggest that they, similar to *Dux*, may be released from perinucleolar heterochromatin and repression (Xie et al., 2022). In human ESCs, chromosome 19 (Chr19), which is enriched for ZGA genes, associates with the nucleolus (Zhang et al., 2024). Upon conversion to eight-cell-like cells, a model of the eight-cell human embryo (Mazid et al., 2022; Taubenschmid-Stowers et al., 2022; Yu et al., 2022), Chr19 moves away from the nucleolus, and several Chr19 ZGA genes are expressed. However, some studies have found that genes moving away from the nucleolus do not immediately become expressed; rather, relocation seems to prime their later activation (Bersaglieri et al., 2022; Gupta et al., 2024 preprint). Conversely, RNA polymerase II transcription can still occur at some nucleolar-proximal regions (Goronzy et al., 2022). Therefore, regulation of gene expression by localisation at the nucleolus may comprise multiple mechanisms, likely specific to particular loci and developmental contexts. Unbiased identification of all such nucleolar-regulated regions in early development will require the development of a genome-wide, low-input method to identify nucleolar associated DNA.

### Discovering factors responsible for tethering chromatin to the nucleolus

Although NADs have now been identified in several cell types, it is still unclear how these regions are tethered to the nucleolus. In addition to NORs themselves, regions in linear proximity to NORs may be assumed to associate with nucleoli by a ‘dragging’ effect of linear distance (Kalmarova et al., 2007). For regions unrelated to NORs, it is likely that specific active mechanisms must recruit DNA loci to the nucleolus. In somatic cells, several studies have identified factors whose depletion disrupts nucleolar heterochromatin. In addition to the example of TIP5 discussed above (Guetg et al., 2010; Santoro et al., 2010), the N-terminal domain of the p150 subunit of the histone-depositing CAF1 complex is responsible for the association of several NAD regions with the nucleolus, via its interaction with several nucleolar proteins (Smith et al., 2014). ZNF274, a Krüppel-associated box (KRAB)-containing zinc-finger protein (KZFP) with a SCAN domain, tethers clusters of lineage-specific genes to the nucleolus in HEK 293T cells, and this tethering is dependent on the SCAN domain and interactions with nucleolar proteins (Begnis et al., 2024).

Another factor that has been implicated in the organisation of nucleolar heterochromatin is NPM1, a nucleolar phosphoprotein found in the outermost layer of the nucleolus. In mouse ESCs, NPM1 binds NADs and mediates H3K9me2 deposition via G9a (Gupta et al., 2024 preprint). Knockdown of NPM1 reduces the H3K9me2-marked chromatin at the nucleolus, but H3K9me3-marked major satellite sequences associated with the nucleolus are unaffected, suggesting multiple tethering mechanisms are at play for different NAD subsets (Gupta et al., 2024 preprint). NPM1 additionally interacts with the heterochromatin factor DOT1L in N2a mouse neuroblast cells, where NPM1 knockdown results in loss of heterochromatin organisation around the nucleolus (Izzo et al., 2023).

While several factors involved in tethering DNA to the nucleolus have now been identified, unanswered questions remain, particularly *in vivo*. Given the enrichment of somatic nucleolar factors at NPBs begins in the late two-cell stage, it remains to be determined what mechanisms dictate DNA arrangement around NPBs in the zygote.

It is not yet known how stably DNA associates with the NPB, but at the late two-cell stage, the major satellite regions move away to form chromocentres, implying dynamic shifting in NADs throughout pre-implantation development. The factors responsible for recruiting and tethering DNA at either the NPB and/or the nucleolus of pre-implantation embryos are largely unknown. However, a recent study has implicated the interaction between a nucleolar lncRNA, long nucleolus-specific lncRNA (LoNA) and NPM1 in nucleolar maturation in two-cell embryos. The loss of LoNA displaces NPM1 from the NPBs and results in embryonic arrest at the two-cell stage (Li et al., 2023). While not explicitly addressing whether DNA tethering at the nucleolus is under the control of NPM1 in two-cell embryos, this study gives a hint that the role of NPM1 may begin at this stage, concomitant with the onset of nucleolar maturation and ZGA.

Another layer of complexity to NAD tethering is the question of sequence specificity. With the exception of ZNF274 (Begnis et al., 2024), which has a specific binding motif enriched at its target DNA sequences, a sequence-specific mechanism for NAD recruitment by tethering proteins remains elusive. Similar to LoNA, the finding that repetitive RNAs, such as those from TIP5/pRNA and LINE1 elements, also tether nucleolar proteins to chromatin in mouse and human, points to an important role for RNA in nucleolar association (Guetg et al., 2010; Lu et al., 2020; Mayer et al., 2006; Percharde et al., 2018; Santoro et al., 2010; Zhang et al., 2024). This is an especially fascinating question in the context of cell-type specificity in NAD selection (Bersaglieri et al., 2022; Bizhanova et al., 2020). It is possible that other RNAs or ZFPs not yet studied may have specificity to the nucleolus, and these factors may, by their own expression dynamics, lend cell-type specificity to NAD selection. Finally, NAD/LAD and LAD only display differences in histone modification, gene density and replication timing. Are these differences instructive in determining whether a locus is NAD/LAD or NAD only, or could sequence-specific tethering mechanisms also be responsible?

## Conclusions

In summary, it is increasingly apparent that the importance of the nucleolus during development is not attributable solely to its role in ribogenesis. Here, we have highlighted nucleolar chromatin as one important facet. New technologies have shed light on nucleolar heterochromatin in a variety of cellular contexts, highlighting the dynamic nature of this genomic compartment in different cell types. An outstanding challenge in the field is now to extend the characterisation of nucleolar-associated DNA to low-input, single-cell and *in vivo* contexts, and define underlying mechanisms. These future directions will help to understand the role of the nucleolar chromatin in development, aging and disease (Box 1).
